# Sensitivity of SARS-CoV-2 Detection With Nasopharyngeal Swabs

**DOI:** 10.3389/fpubh.2020.593491

**Published:** 2021-01-26

**Authors:** Bianca Clerici, Antonio Muscatello, Francesca Bai, Donatella Pavanello, Michela Orlandi, Giulia C. Marchetti, Valeria Castelli, Giovanni Casazza, Giorgio Costantino, Gian Marco Podda

**Affiliations:** ^1^Divisione di Medicina Generale II, ASST Santi Paolo e Carlo, Dipartimento di Scienze della Salute, Università degli Studi di Milano, Milan, Italy; ^2^Unità Operativa Complessa di Malattie Infettive, IRCCS Fondazione Ca' Granda, Ospedale Maggiore Policlinico, Milan, Italy; ^3^Dipartimento di Malattie Infettive, ASST Santi Paolo e Carlo, Università degli Studi di Milano, Milan, Italy; ^4^U.O.C Pronto Soccorso e Medicina D'Urgenza, IRCCS Fondazione Ca' Granda, Ospedale Maggiore Policlinico, Università degli Studi di Milano, Milan, Italy; ^5^Dipartimento di Scienze Biomediche e Cliniche “L. Sacco”, Università degli Studi di Milano, Milan, Italy

**Keywords:** COVID-19, sensitivity, swab analysis, diagnosis, false negative (FN)

## Abstract

**Background:** SARS-CoV-2-infected subjects have been proven contagious in the symptomatic, pre-symptomatic and asymptomatic phase. The identification of these patients is crucial in order to prevent virus circulation. No reliable data on the sensitivity of nasopharyngeal swabs (NPS) are available because of the lack of a shared reference standard to identify SARS-CoV-2 infected patients. The aim of our study was to collect data on patients with a known diagnosis of COVID-19 who underwent serial testing to assess NPS sensitivity.

**Methods:** The study was a multi-center, observational, retrospective clinical study with consecutive enrollment. We enrolled patients who met all of the following inclusion criteria: clinical recovery, documented SARS-CoV-2 infection (≥1 positive rRT-PCR result) and ≥1 positive NPS among the first two follow-up swabs. A positive NPS not preceded by a negative nasopharyngeal swab collected 24–48 h earlier was considered a true positive. A negative NPS followed by a positive NPS collected 24–48 h later was regarded as a false negative. The primary outcome was to define sensitivity of SARS-CoV-2 detection with NPS.

**Results:** Three hundred and ninety three NPS were evaluated in 233 patients; the sensitivity was 77% (95% CI, 73 to 81%). Sensitivity of the first follow-up NPS (*n* = 233) was 79% (95% CI, 73 to 84%) with no significant variations over time. We found no statistically significant differences in the sensitivity of the first follow-up NPS according to time since symptom onset, age, sex, number of comorbidities, and onset symptoms.

**Conclusions:** NPS utility in the diagnostic algorithm of COVID-19 should be reconsidered.

## Background

Severe acute respiratory syndrome coronavirus 2 (SARS-CoV-2) is responsible of coronavirus disease 2019 (COVID-19) (1). SARS-CoV-2-infected subjects have been proven contagious in the symptomatic, pre-symptomatic, and asymptomatic phase (2, 3). The identification of these patients is crucial in order to prevent virus circulation. The ideal diagnostic test should be easily accessible, not invasive, with quick results and possibly cheap. Presently, clinicians rely on real time reverse transcription polymerase chain reaction (rRT-PCR) tests performed on various biological specimens (4). Lower respiratory tract specimens display the highest sensitivity (5), however their collection is not feasible at large scale. The most accessible diagnostic test is rRT-PCR on upper respiratory tract samples, such as nasopharyngeal swabs. Assuming a 100% specificity (6), no reliable data on the sensitivity of nasopharyngeal swabs are available because of the lack of a shared reference standard to identify SARS-CoV-2 infected patients. In fact, rRT-PCR-based tests imply known pre-analytical and analytical vulnerabilities (7). Composite reference standards including clinical and radiological features have been used (8, 9), none of which are however pathognomonic of COVID-19. RNA-positivity of biological specimens has been shown to outlast symptom resolution (10). For these reasons we decided to collect data on patients with a known diagnosis of COVID-19 who underwent serial testing to assess nasopharyngeal swab sensitivity.

## Methods

### Study Design and Population

The study was a multi-center, observational, retrospective clinical study with consecutive enrollment. Participating centers included IRCCS Fondazione Ca' Granda Ospedale Maggiore Policlinico and ASST Santi Paolo e Carlo, Università degli Studi di Milano, both based in Milan. All patients who sequentially referred to the two participating centers for follow-up outpatient testing with nasopharyngeal swabs between 05/03/2020 and 20/05/2020 were screened for enrollment. We enrolled patients who met all of the following inclusion criteria: clinical recovery (apyrexia and no need for supplemental oxygen therapy for 3 consecutive days), documented SARS-CoV-2 infection (≥1 positive rRT-PCR result) and ≥1 positive nasopharyngeal swab among the first two follow-up swabs. We excluded patients whose first two follow-up swabs delivered negative results (viral clearance).

### Nasopharyngeal Swab Technique

All patients, once clinically recovered, were to undergo the first follow-up swab after 14 days since hospital discharge. In case of a positive result, the test was to be repeated after 7 days; in case of a negative result, a second swab was performed 24–48 h later. If this was also negative, patient isolation was ended (11); if positive, the test was to be repeated after 7 days. No other kind of respiratory specimen was collected for follow-up purposes. Nasopharyngeal swabs were performed, stored and delivered to the testing laboratory as recommended by the CDC and ECDC. Nasopharyngeal swabs were performed following a standardized procedure (12). Briefly, GeneFinder™ COVID-19 PLUS RealAmp Kit has been used for detection of SARS-CoV-2 virus through reverse Transcription and Real-Time Polymerase Chain Reaction from RNA extracted from nasopharyngeal swab (ELITe InGenius®system; ELITechGroup, Puteaux, France). The extraction volume was 200 μL. One-Step Reverse Transcription Real-Time polymerase chain reaction is used to confirm the presence of COVID-19 by amplification of RdRp, E, and N genes. The cut-off Ct value of GeneFinder COVID-19 Plus RealAmp Kit (ELITechGroup, Puteaux, France) assay is 40 and the analytical sensitivity of the assay is 1 copy/ μL.

### Definitions

**Figure 1:**
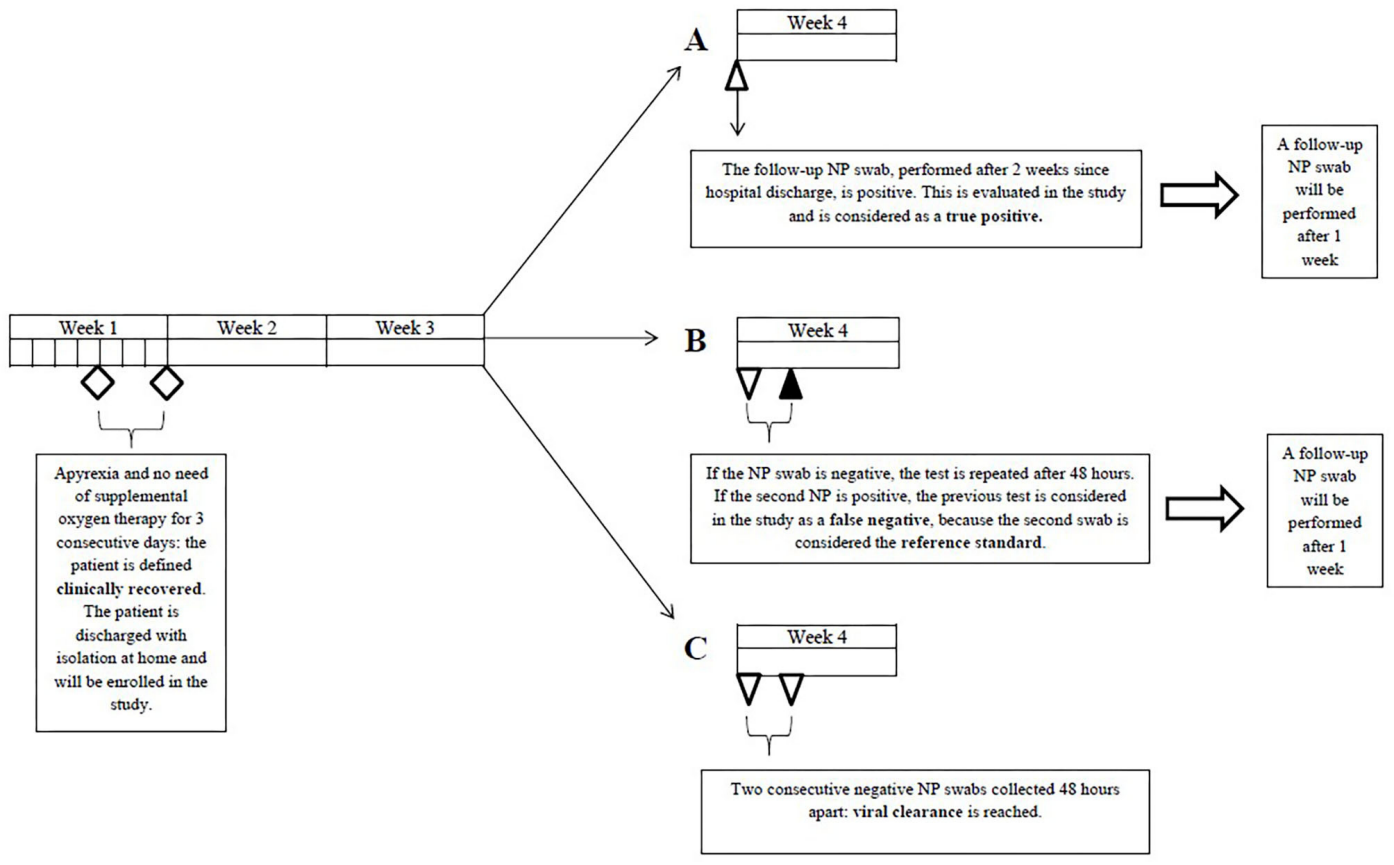
Definition of true positive and false negative nasopharyngeal swabs. NP, nasopharyngeal.

A positive nasopharyngeal swab not preceded by a negative nasopharyngeal swab collected 24–48 h earlier was considered a true positive (TP). A negative nasopharyngeal swab followed by a positive nasopharyngeal swab collected 24–48 h later was regarded as a false negative (FN) (Figure 1).

### Outcomes

The primary outcome was to define sensitivity of SARS-CoV-2 detection with nasopharyngeal swabs. The secondary outcome was to evaluate nasopharyngeal swab sensitivity over time and the association between the sensitivity of the first nasopharyngeal follow-up swab and the patient's age, sex, comorbidities, and onset symptoms.

### Statistical Analysis

We estimated that the enrollment of 230 patients with documented SARS-CoV-2 infection would enable to estimate a 63% sensitivity with an acceptable precision, as quantified by the 95% CI (56 to 69%). Patient data have been recorded on Microsoft Excel and analyzed with STATA 15 (StataCorp. 2017. Stata Statistical Software: Release 15. College Station, TX: StataCorp LLC). Data were expressed as means ± standard deviation (SD) or medians with interquartile ranges (IQR) as appropriate and sensitivities were compared using the Chi-squared test.

## Results

### Patients

Seven hundred and six patients were screened for enrollment after referral to the two participating centers for serial nasopharyngeal swab outpatient testing between 05/03/2020 and 20/05/2020. Four hundred and seventy-three patients reached viral clearance after the first two follow-up nasopharyngeal swabs, and were therefore excluded. Two hundred and thirty-three patients met all inclusion criteria. All patients had ≥1 positive rRT-PCR test collected at the time of diagnosis of COVID-19. All patients received the first follow-up swab once clinically recovered, in most cases 14 days after hospital discharge. The patients' median age was 55.3 years (interquartile range, 43.4 to 64.4), 39% of the patients were women, 74% were Caucasian, 15% were Hispanic, 7% were Maghrebian, Middle Eastern or Arab, and 3% were Asian. The ethnicity of 4 patients was unknown. Clinical data were available for 222 patients. Forty-nine patients (22%) had no comorbidities, while 169 (78%) had ≥1. One hundred and eighty-seven patients (84%) had pneumonia. The most frequent onset symptoms were fever (96%), cough (80%), dyspnea (47%), fatigue (45%), ageusia (41%), and anosmia (34%). All patients underwent ≥1 follow-up swab. The median number of TP and FN nasopharyngeal swabs per patient was 1 (range, 1 to 6). Of the 233 patients included in our analysis, 182 (78%) reached viral clearance. At the end of the study period data collection was still ongoing for the remaining 51 (22%). The median time to viral clearance was 45.0 days (interquartile range, 38.0 to 52.7) since symptom onset.

### Sensitivity

**Figure 2:**
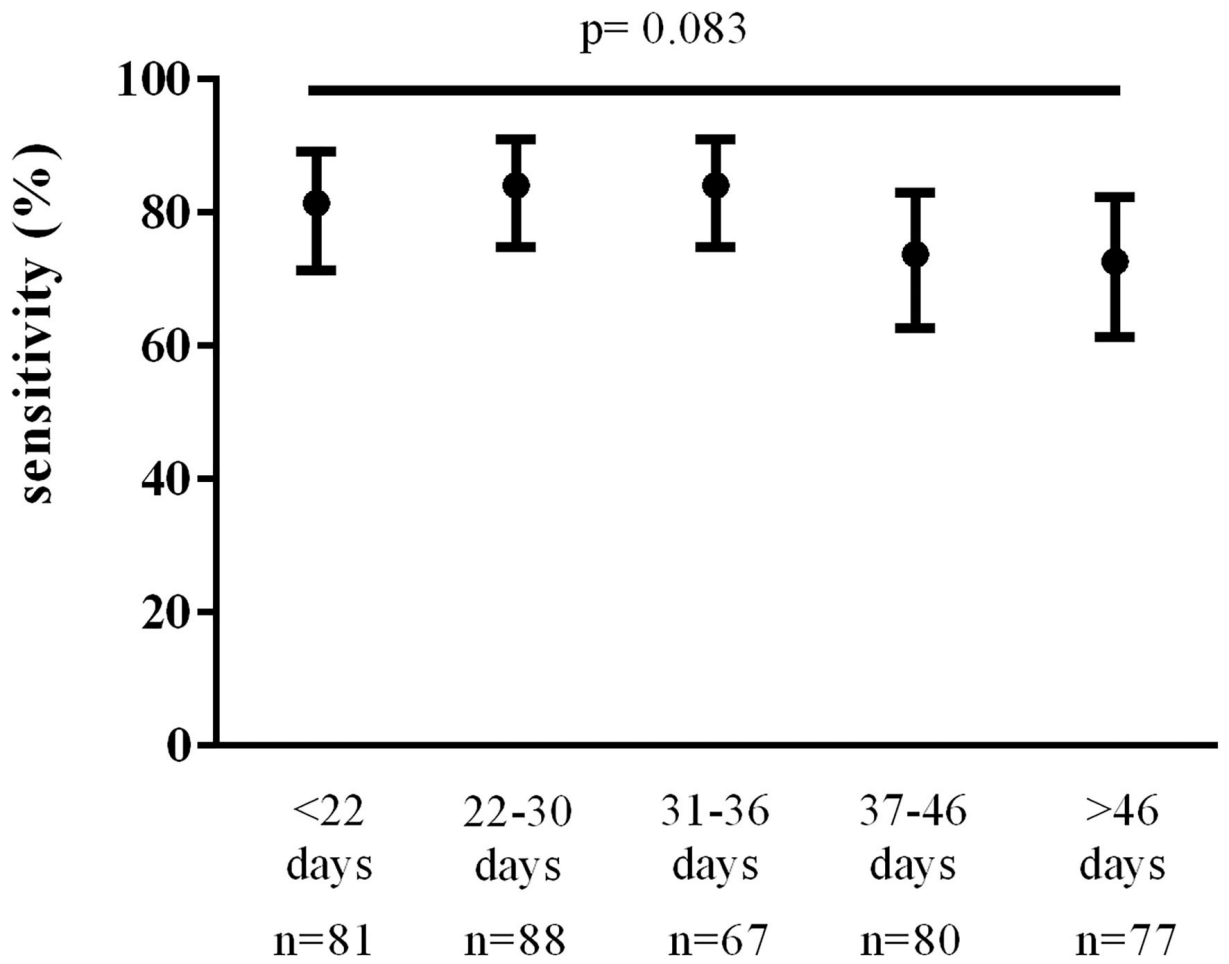
Nasopharyngeal swab sensitivity over time since symptom onset. Data were analyzed using the Chi-square test.

The total number of TP and FN follow-up nasopharyngeal swabs performed in our patient population was 393. Total TP swabs were 303; total FN swabs were 90. Of the 233 first follow-up swabs of our data set, 184 were TP and 49 were FN. Overall nasopharyngeal swab sensitivity was 77% (95% CI, 73 to 81%). Sensitivity of the first follow-up nasopharyngeal swabs (*n* = 233) was 79% (95% CI, 73 to 84%) with no significant variations over time (Figure 2). We found no statistically significant differences in the sensitivity of the first follow-up nasopharyngeal swab according to time since symptom onset, age, sex, number of comorbidities, and onset symptoms.

## Discussion and Conclusions

Because of the unavailability of a shared reference standard for COVID-19 diagnosis there are no reliable data on nasopharyngeal swab sensitivity. We decided to assess nasopharyngeal swab sensitivity in patients with known SARS-CoV-2 infection, based on the presence of symptoms and of ≥1 positive rRT-PCR test, who underwent serial testing. In our patient population sensitivity was 77% (95% CI, 73 to 81%). Wang et al. evaluated SARS-CoV-2 detectability in different biological specimens in a similar cohort of COVID-19 patients and found a nasopharyngeal swab sensitivity of 63% (5); however, this result was based on 8 samples. Kucirka et al. pooled data from 7 relevant studies on both nasal and throat swabs, and found that the probability of a false negative result was as high as 21% even at the optimal testing window (3 days after symptom onset) (13). Two of the 7 analyzed studies included both rRT-PCR confirmed cases and probable cases, identified through clinical criteria alone.

Our sensitivity assessment might have been overestimated. Firstly, most of the patients included in our study had ≥1 positive nasopharyngeal swab at time of diagnosis. Secondly, positive follow-up nasopharyngeal swabs weren't followed by a second swab 24–48 h later, unlike negative swabs; this might have led to the underestimation of the number of FN nasopharyngeal swabs. On the other hand, we didn't perform further nasopharyngeal swabs once viral clearance was reached; this, too, might have contributed to the underestimation of total FN swabs.

Our study has some limitations. The fact that nasopharyngeal swab sensitivity varies throughout disease course limits the external validity of our findings. Although we might have overestimated true nasopharyngeal swab sensitivity for the aforementioned reasons, sensitivity may be likely higher at the beginning of the disease, when viral shedding is greater. We did not investigate the association between the antiviral treatment received during hospitalization and sensitivity of the first follow-up swab. Lastly, as recommended by the WHO (11), we didn't perform further rRT-PCR-based tests after viral clearance was reached. We cannot exclude that subsequent tests might have shown the recurrence of positive nasopharyngeal swabs at least in some patients. Finally, it is possible that the sensitivity values of the nasopharyngeal swabs are influenced by pre-analytical and/or analytical variables; therefore, we cannot exclude that different RT-PCR methods may be sources of variability in the sensitivity of the nasopharyngeal swabs.

In conclusion, in our large cohort of COVID-19 patients, sensitivity of SARS-CoV-2 detection with rRT-PCR on nasopharyngeal swabs was 77% (95% CI, 73 to 81%). For the purposes of our study, we assumed specificity to be 100%. It is however safe to say that a positive nasopharyngeal swab indicates SARS-CoV-2 infection. Conversely, with consideration of the 77% sensitivity we found, a negative result alone cannot rule out infection when this is suspected on clinical or epidemiological grounds. This has led to serial retesting in clinical practice. We think that such a strategy should not be encouraged since it is time-consuming, requires complex organization and leads to facilities overcrowding and delay in treatment initiation. An alternative diagnostic strategy is urgently needed.

## Data Availability Statement

The raw data supporting the conclusions of this article will be made available by the authors, without undue reservation.

## Ethics Statement

The studies involving human participants were reviewed and approved by Comitato Etico Milano Area. All subjects signed a written informed consent to personal data treatment, which allowed the anonymous use of clinical data for research purposes.

## Author Contributions

AM, FB, DP, MO, GM, and VC provided clinical data and revised the manuscript. GMP conceived the study and wrote the manuscript. BC wrote and edited the manuscript. GCo conceived and designed the study and revised the manuscript. GCa performed the analysis. All authors contributed to the article and approved the submitted version.

## Conflict of Interest

The authors declare that the research was conducted in the absence of any commercial or financial relationships that could be construed as a potential conflict of interest.
